# Honokiol-Mediated Mitophagy Ameliorates Postoperative Cognitive Impairment Induced by Surgery/Sevoflurane via Inhibiting the Activation of NLRP3 Inflammasome in the Hippocampus

**DOI:** 10.1155/2019/8639618

**Published:** 2019-02-24

**Authors:** Ji-Shi Ye, Lei Chen, Ya-Yuan Lu, Shao-Qing Lei, Mian Peng, Zhong-Yuan Xia

**Affiliations:** ^1^Department of Anesthesiology, Renmin Hospital of Wuhan University, Wuhan, 430060 Hubei, China; ^2^Department of Anesthesiology, Zhongnan Hospital of Wuhan University, 169, Donghu Road, Wuhan, 430071 Hubei, China

## Abstract

**Background:**

The potential mechanism of postoperative cognitive impairment is still largely unclear. The activation of NLRP3 inflammasome had been reported to be involved in neurodegenerative diseases, including postoperative cognitive change, and is closely related to mitochondrial ROS and mitophagy. Honokiol (HNK) owns multiple organic protective effects. This study is aimed at observing the neuroprotective effect of HNK in postoperative cognitive change and examining the role of HNK in the regulation of mitophagy and the relationship between these effects and NLRP3 inflammasome activation in mice induced by surgery/anesthesia.

**Methods:**

In this study, mice were divided into several groups: control group, surgery group, surgery+HNK group, and surgery+HNK+3-methyladenine (3-MA) group. Hippocampal tissue samples were harvested and used for proinflammatory cytokines, mitochondrial ROS, and malondialdehyde (MDA) assay. The process of mitophagy and the activation of NLRP3 inflammasome were observed by Western blot, immunohistochemistry, and transmission electron microscopy.

**Results:**

The results showed that HNK treatment obviously recovered the postoperative decline and enhanced the expressions of LC3-II, Beclin-1, Parkin, and PINK1 at protein levels after surgery/sevoflurane treatment, which are both an autophagy marker and a mitophagy marker. In addition, HNK attenuated mitochondrial structure damage and reduced mtROS and MDA generation, which are closely associated with NLRP3 inflammasome activation. Honokiol-mediated mitophagy inhibited the activation of NLRP3 inflammasome and neuroinflammation in the hippocampus. Using 3-MA, an autophagy inhibitor, the neuroprotective effects of HNK on mitophagy and NLRP3 inflammasome activation were eliminated.

**Conclusion:**

These results indicated that HNK-mediated mitophagy ameliorates postoperative cognitive impairment induced by surgery/sevoflurane. This neuroprotective effect may be involved in inhibiting the activation of NLRP3 inflammasome and suppressing inflammatory responses in the hippocampus.

## 1. Introduction

Surgery/anesthesia is often an inevitable medical intervention in many patients during hospitalization. Postoperative cognitive decline (POCD) describes a cluster of cognitive behavior abnormalities including a relative drop in learning and memory performance on a set of neuropsychological tests from before to after surgery [1]. Dissecting the mechanisms of POCD becomes important, not only because it is a pathophysiological problem that we do not yet illuminate completely but also because it is a common postoperative complication that affects the quality of the patients' daily life and long-term outcome [2]. Like Alzheimer's disease (AD) and other neurodegenerative diseases, the potential pathophysiological mechanism of POCD may also be involved in neuroinflammation, oxidative stress, blood-brain barrier dysfunction, and apoptosis [2–6].

In recent years, several lines of studies have focused their attention on inflammasomes, which are essential components of the innate immune system and play a pivotal role in pro- or anti-inflammatory homeostasis [6–8]. Inflammasomes are intracellular multiprotein complexes that drive the activation of inflammatory responses. Among all types of inflammasomes, such as NLRP1, NLRP3, NLRC4, and AIM2, NLRP3 is the most studied one, especially in the central neural system [9, 10]. NLRP3 inflammasome activation could recruit and activate Caspase-1, leading to the secretion of mature IL-1*β* and IL-18 and the initiation of a novel form of cell death named pyroptosis [11]. Emerging evidence showed that NLRP3 inflammasomes could be identified in microglia, astrocytes and neurons, which induced neuroinflammation in a series of neurodegenerative diseases [7, 8, 12, 13]. So, in the surgery/sevoflurane model, we can observe whether the NLRP3 inflammasome was activated and could influence the neurological outcome. Moreover, a set of researches have also uncovered that the high levels of reactive oxygen species (ROS) are a common step that is essential for the formation of NLRP3 inflammasome [14]. Mitophagy, an autophagic process that specifically autophagically degrades damaged and free radical-generating mitochondria, regulates the mitochondrial homeostasis and cellular survival [15]. As mitophagy is impaired, the overaccumulation of mitochondrial ROS from the damaged mitochondria could induce NLRP3 inflammasome activation and lead to the inflammatory cascade [16]. Therefore, recent studies have demonstrated that regulation of autophagy/mitophagy may be a novel target for NLRP3-dependent proinflammatory responses in CNS disorders and metabolic inflammation.

Honokiol (HNK) (2-(4-hydroxy-3-prop-2-enyl-phenyl)-4-prop-2-enyl-phenol) is a bioactive compound obtained from *Magnolia grandiflora*, a species of magnolia common in Japan, which possesses multiple properties including antitumor, antiarrhythmic, antithrombocytic, anti-inflammatory, antiangiogenesis, and antioxidative activities in vivo and in vitro [17–21]. In previous studies, we found that honokiol have the protective effect on amyloid *β* oligomer-induced Alzheimer's disease in mice via attenuating mitochondrial apoptosis [22]. And in our preliminary studies (unpublished), honokiol could also ameliorate the oxidative stress and neuroinflammation in mice induced by surgery/anesthesia. However, the influence of HNK on mitophagy and its relationship with the NLRP3 inflammasome in surgery/sevoflurane models are still unknown.

In the present study, to improve the understanding of the neuroprotective effect of HNK in POCD, we observed the role of HNK in the regulation of mitophagy and the relationship between these effects and NLRP3 inflammasome activation in mice induced by surgery/anesthesia.

## 2. Materials and Methods

### 2.1. Animals

The animal use and care protocols were approved by the Animal Ethics Committee of Zhongnan Hospital of Wuhan University, Hubei, China. 4-month-old adult female C57BL/6J mice weighing 20–25 g were purchased from the Beijing Vital River Laboratory. All animals were acclimatized to the laboratory condition for at least 7 days prior to use. The environment of animal housing was under a 12-h/12-h light/dark cycle at 25°C and 50%-65% humidity with free access to food and water.

### 2.2. Experimental Group and Treatment

Animals were divided into six groups: (1) control group; (2) surgery+vehicle group (mice treated with vehicle for 1 week and underwent surgical operation); (3) surgery+HNK group (mice treated with HNK and underwent surgical operation); (4) surgery+HNK+3-MA group (mice treated with HNK and 3-MA and underwent surgical operation); (5) control+HNK group (control mice pretreated with HNK); and (6) control+HNK+3-MA group (control mice pretreated with HNK and 3-MA). Both honokiol and 3-MA were obtained from Sigma–Aldrich (St. Louis, MO, USA). They were dissolved in 0.5% dimethyl sulfoxide (DMSO). The animals received daily intraperitoneal injections of HNK at a dose of 10 mg/kg, 3-MA at a dose of 2 mg/kg, or DMSO for 7 days before the surgery. The control group mice received daily intraperitoneal injections of equal volume (0.5 mL) 0.5% DMSO for 7 days. Drug dosages were selected based on data from previous studies [23, 24] and preliminary experiments.

**Figure 1:**
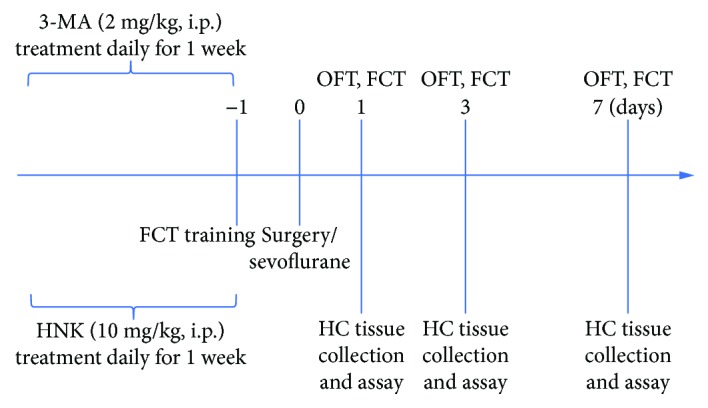
The study plan and schematic diagram of the experimental timeline. HNK: honokiol; FCT: fear conditioning test; OFT: open-field test; HC: hippocampus.

After fear conditioning training for 1 day, all animals received surgery/sevoflurane exposure. The open-field test (OFT) was achieved 20 min before each test phase of fear conditioning at postoperative 1, 3, and 7 days. And then the mice performed the fear conditioning test (FCT). After each test, the OFT and the FCT were washed with 75% ethanol to eliminate olfactory cues. The mice were sacrificed for biochemistry detection 1 h after all behavioral evaluations. The detailed study plan is graphically described in Figure 1.

### 2.3. Anesthesia and Surgery

The mice were subjected to abdominal exploratory surgery under general anesthesia via inhaling sevoflurane. Briefly, animals were anesthetized with 5% sevoflurane and maintained with 3% sevoflurane carried by 5 L/min oxygen. To avoid carbon dioxide retention and deep anesthesia, the concentration of sevoflurane and carbon dioxide was continuously monitored by an anesthesia gas monitor (Dräger Medical GmbH, Lübeck, Germany). Under spontaneous breathing, a 3 cm midline abdominal incision was made in the midline and the abdominal organs were explored gently with sterilized gauze. Using 9/0 Prolene sutures (Ethicon, USA), the incision was closed neatly. Then, a 0.2% lidocaine solution was administered subcutaneously for postoperative analgesia. All processes lasted approximately 50 mins. And a heat pad was utilized to keep the rats' body temperature at ~37°C during the surgery. At the end of the operation, 0.5 mL saline was administered by intraperitoneal injection for fluid supplementation. The mice of the control group did not receive the surgical operation.

### 2.4. Open-Field Test

To assess the effect of surgery/sevoflurane on locomotor activity of the mice, we performed the open-field test in an open-field chamber (60 × 60 × 40 cm) under dim light. The floor of a plastic transparent box was divided in 16 equal-sized squares. The center zone was the four squares in the center and the periphery was the 12 squares along the walls. Each animal was put into the center of the box and was permitted to travel for 5 min. The total travelled distance and the amount of time spent in the center zone were recorded via the ANY-maze animal tracking system software (Xinruan, Shanghai, China).

### 2.5. Fear Conditioning Test

Fear conditioning tests (FCT) were performed to assess memory in animals. The freezing behavior times of the mice reflect the capacity of learning and memory. Based on previously published studies, FCT includes two parts: a training phase at 1 day before surgical operation and a test phase at postoperative 1 day and 3 days.

In the training phase, the mice were trained for fear conditioning to establish the long-term memory. All animals were in the same training session and allowed to adapt to the environments (context) for 120 seconds, followed by six cycles of conditional-unconditional stimuli. A cycle of conditional-unconditional stimuli was then applied as a 20 s, 80 dB tone (conditional stimuli)—30 s delay—5 s, 0.75 mA electrical foot shock (unconditional stimuli). The cycles of conditional-unconditional stimuli were separated by random intervals from 45 to 60 seconds.

The context test, which reflects hippocampal-dependent memory, is the part of FCT. 1, 3, and 7 days after surgical operation, all the mice were placed back into the original conditioning box for 5 min, where no tone and no shock were produced. The percentage of time spent not moving (percentage freezing time) was captured and recorded by a video camera mounted above the center of the pool.

### 2.6. Apoptosis Detection in the Hippocampus

After behavioral tests, the mice were deeply anesthetized with pentobarbital sodium (50 mg/kg). To clear the blood in the circulatory system, the mice underwent a thoracotomy for transcardial perfusion with 0.9% NaCl, followed by 4% paraformaldehyde. Then, the fixed brain was removed and postfixed in 4% paraformaldehyde overnight at 4°C, then embedded in paraffin. Terminal deoxynucleotidyl transferase-mediated dUTP nick-end labeling (TUNEL) staining was performed according to the manufacturer's instruction (Roche, South San Francisco, CA, USA). Neuronal apoptosis was analyzed in the hippocampus sections. The quantification of TUNEL-positiveneurons in the hippocampal CA1 and DG was performed by a pathologist in 3 fields per slide randomly at 200× magnification.

### 2.7. Immunohistochemistry

The mice were deeply anesthetized with pentobarbital sodium (50 mg/kg) and perfused intracardially with saline followed by 4% paraformaldehyde. Then, the fixed cerebral tissues were postfixed in 4% paraformaldehyde at 4°C for 24 h and embedded in paraffin. The brain sections (4 *μ*m thickness) were incubated overnight at 4°C with a primary antibody against NLRP3. After washing carefully in PBS for about 15 min, the sections were then incubated with a second antibody (1 : 200) and AB work solution (Vector Laboratories, Burlingame, CA, USA) for 30 min at room temperature. DAB solution was used to visualize the staining of NLRP3.

### 2.8. Transmission Electron Microscopy

1 mm^3^ tissue from the brain tissues from each group of mice was cut and fixed with a solution containing 2% (*v*/*v*) glutaraldehyde and 4% formaldehyde in 0.1 M sodium cacodylate, pH 7.4, for 15 min at room temperature. The fixed samples were then treated at the Wuhan Institute of Virology, Chinese Academy of Sciences, for further steps as previously described. Images were acquired using a Tecnai G^2^ 20 TWIN transmission electron microscope.

### 2.9. Western Blot

After behavior tests, the mice were killed and their hippocampal tissues were harvested. Total proteins were separated by SDS-PAGE after denaturation and transferred onto polyvinylidene difluoride (PVDF) membranes. After blocking with 5% skim milk, the membranes were incubated with rabbit anti-mouse monoclonal antibodies against NLRP3 (1 : 200, Novus, USA); Caspase-1 and ASC (1 : 200, Santa Cruz, CA, USA); PINK1, Parkin, and Beclin-1 (1 : 500, 1 : 1000, and 1 : 500, respectively, Abcam, Cambridge, UK); LC-3 (1 : 10000; Cell Signaling, USA); and GAPDH (1 : 1000, Abcam, Cambridge, UK) overnight at 4°C with shaking. Then, the membranes were washed in TBST. After that, the membranes were washed and incubated with secondary antibody anti-rabbit IgG (1 : 2000, Santa Cruz, CA, USA) for 1.5 h at room temperature. All blots were scanned and analyzed using the Odyssey Infrared Imaging System (LI-COR Biosciences).

### 2.10. Isolation of Mitochondria from the Hippocampus

The intact mitochondria of the hippocampal region were isolated from the fresh hippocampus by using a tissue mitochondria isolation kit (Beyotime, China). Briefly, the hippocampus tissues were homogenized in an ice-cold buffer as previously described [25]. And then, the homogenate was centrifuged at 6000×g at 4°C for 5 min. After that, the collected supernatant was then centrifuged at 11000×g at 4°C for 10 min to obtain a mitochondrial pellet. Then, the pellet was stored as mitochondria and suspended in the mitochondrial storage fluid provided in the kit.

### 2.11. Enzyme-Linked Immunosorbent Assay (ELISA)

We detected the hippocampal levels of IL-1*β* and TNF-*α* at 24 h after isoflurane exposure by ELISA kits (R&D Systems, DY401) following the protocols provided by the manufacturer (Abcam). Readings were normalized to the amount of a standard protein.

### 2.12. Statistical Analysis

All statistical analyses were performed with SPSS12 (SPSS Inc., Chicago, IL) and GraphPad Prism 5 (GraphPad, San Diego, CA). All data are presented as mean ± standard error of the mean (SEM). Statistical analysis of differences between groups was performed by using a one-way ANOVA followed by a SNK test for post hoc comparisons; *p* < 0.05 was considered statistically significant.

## 3. Results

### 3.1. HNK Enhanced Cognitive Recovery in Surgery/Sevoflurane-Treated Mice

Previous and our preliminary studies showed that surgery/anesthesia (sevoflurane or isoflurane) induced behavioral and cognitive impairment in mice.

To evaluate the postoperative cognitive decline in mice induced by surgery/sevoflurane and the protective effect of HNK, we assessed locomotor activity, learning, and memory by using the open-field test and the contextual fear conditioning tests, respectively.

In the open-field test, there are no significant differences in the total distance among the groups at postoperative 1, 3, and 7 days. These data suggested that surgery/sevoflurane, HNK, and 3-MA had no effect on the locomotor activity of the mice.

**Figure 2:**
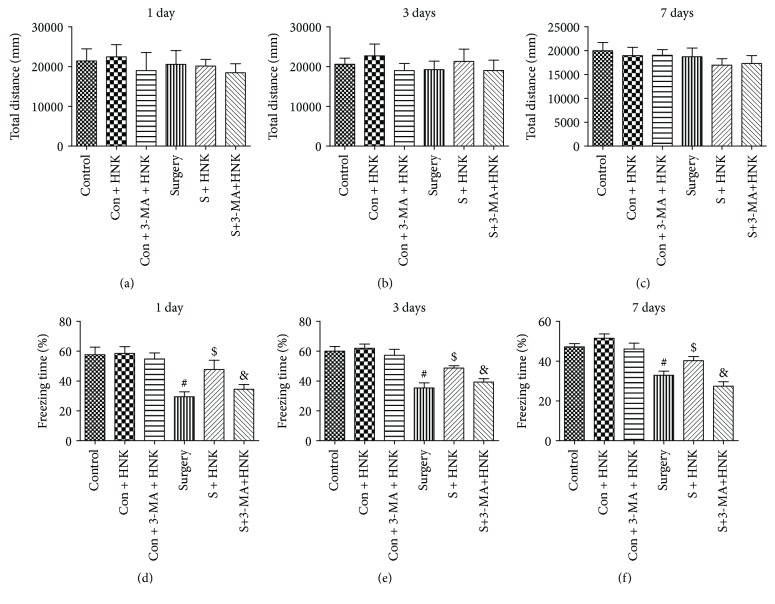
The effects of HNK on cognitive recovery in surgery/sevoflurane-treated mice. (a–c) The total distance traveled during 5 min of open-field exploration at postoperative 1, 3, and 7 days. (d–f) The percentage of freezing time during 5 min in context test (test phase of the FCT) at postoperative 1, 3, and 7 days. The data are presented as the mean ± standard error of the mean for each group (*n* = 6 per group). ^#^*p* < 0.05 versus the control group; ^$^*p* < 0.05 versus the surgery+vehicle group; and ^&^*p* < 0.05 versus the surgery+HNK group.

In the contextual fear conditioning test, there are significant differences among the groups at postoperative 1, 3, and 7 days. Meanwhile, we found that HNK or 3-MA pretreatment alone did not change the cognitive function of the control mice (*p* > 0.05). Besides, we also observed that surgery under sevoflurane anesthesia could reduce the freezing time of the contextual fear response compared with the control group up to postoperative 7 days, indicating the hippocampal impairments with postoperative cognitive decline (*p* < 0.05, Figures 2(d)–2(f)). Notably, HNK prevented the memory impairment from surgical stress and restored freezing behavior, the index for memory retention in mice (*p* < 0.05, Figures 2(d)–2(f)). However, 3-MA, an autophagy and mitophagy inhibitor, abolished the protective effect of HNK (*p* < 0.05, Figures 2(d)–2(f)). These data indicated that HNK may regulate autophagy or mitophagy to provide neuroprotection in postoperative cognitive impairment.

### 3.2. HNK Improved Mitophagy and Reduced the Levels of Mitochondrial ROS in the Hippocampus of Mice Induced by Surgery/Sevoflurane

**Figure 3:**
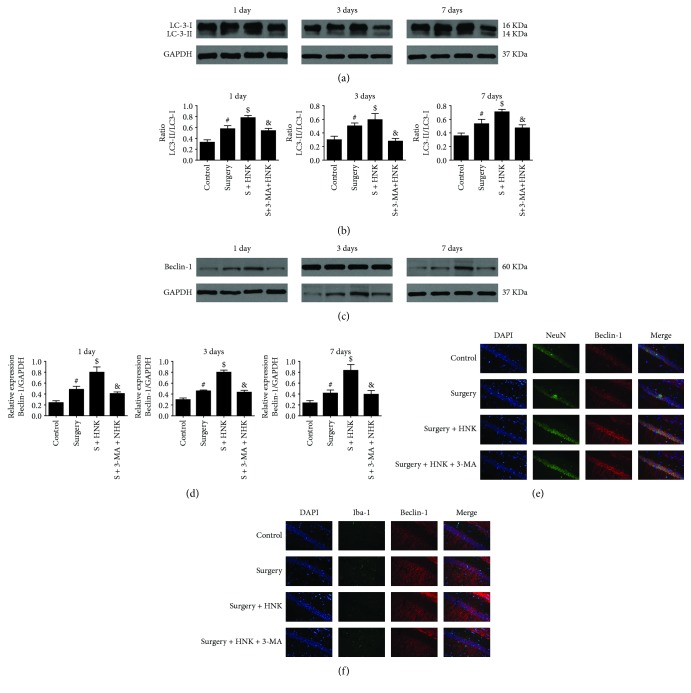
The effects of HNK on autophagy in the hippocampus of mice induced by surgery/sevoflurane. (a–d) The Western blot representative blots of autophagy-related proteins, LC3-II, and Beclin-1 (*n* = 6 per group). (e, f) Representative double immunofluorescence labeling of Beclin-1 with NeuN and Iba-1 in the CA1 at 24 h after surgery/sevoflurane. Scale bar = 50 *μ*m. The data are presented as the mean ± standard error of the mean for each group (*n* = 6 per group). ^#^*p* < 0.05 versus the control group; ^$^*p* < 0.05 versus the surgery+vehicle group; and ^&^*p* < 0.05 versus the surgery+HNK group.

To evaluate the levels of autophagy and mitophagy in mice under surgery operation, we examined the expressions of autophagy and mitophagy biomarkers by Western blotting and immunofluorescence. And ultrastructural morphological changes of the mitochondria were observed by transmission electron microscopy (TEM). Surgery under sevoflurane could upregulate the expression levels of autophagy-related proteins, LC3-II, and Beclin-1, compared with the control group up to postoperative 7 days (*p* < 0.05, Figure 3). Intriguingly, pretreatment with HNK could further enhance the expression of autophagy biomarkers compared with the surgery/sevoflurane group (*p* < 0.05, Figure 3). And 3-MA markedly decreased the HNK-induced autophagy enhancement in mice under surgery/sevoflurane (*p* < 0.05, Figure 3).

Double immunofluorescence suggested that Beclin-1 was colocalized with positive staining of Iba-1 and NeuN (microglia and neuron marker, respectively) in the hippocampus at 1 day after surgery/sevoflurane. These results demonstrated that autophagy activation could occur in the two most common types of neuronal cells of mice induced by surgery/sevoflurane. Meanwhile, the expression of microglial marker Iba-1 was upregulated in the hippocampus of the mice in the surgery group on postoperative 1 day compared to the control group (Figure 3, *p* < 0.05). For autophagy, the expression of Beclin-1 was upregulated in the hippocampus of mice at 1 day after surgery/anesthesia compared to the control group, while HNK pretreatment could further augment the Beclin-1 expression in the hippocampus and 3-MA inhibited these results (Figure 3, *p* < 0.05). However, different treatments had no effect on the NeuN expression (*p* > 0.05).

**Figure 4:**
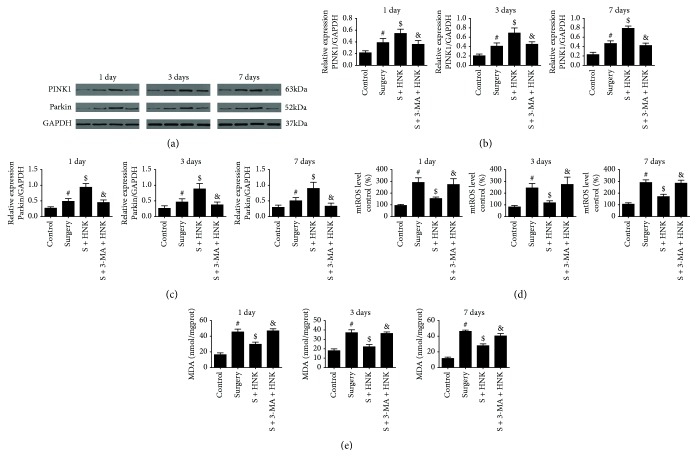
The effects of HNK on mitophagy and oxidative stress in the hippocampus of mice induced by surgery/sevoflurane. (a–c) The Western blot representative blots of mitophagy-related proteins, Parkin, and PINK1 (*n* = 6 per group). (d, e) The oxidative indicators of mtROS levels and MDA in the hippocampus induced by surgery/sevoflurane. The data are presented as the mean ± standard error of the mean for each group. ^#^*p* < 0.05 versus the control group; ^$^*p* < 0.05 versus the surgery+vehicle group; and ^&^*p* < 0.05 versus the surgery+HNK group.

Mitophagy, a process of selective autophagy, removed damaged and superfluous mitochondria from the cell by the Parkin/PINK1 pathway. The mice underwent surgery/sevoflurane and promoted the PINK1 and Parkin protein expression compared with the control group up to postoperative 7 days, whereas HNK pretreatment further improved these expressions (*p* < 0.05, Figures 4(a)–4(c)). Moreover, 3-MA intervention could ameliorate the upregulation of these mitophagy-related proteins (*p* < 0.05, Figures 4(a)–4(c)). Mitochondrial ROS (mtROS) and malondialdehyde (MDA) can also reflect mitochondria function and the degree of oxidative stress. There are significant differences among the groups at postoperative 1, 3, and 7 days (Figures 4(d) and 4(e)). The levels of MDA and mtROS were significantly increased in the surgery/sevoflurane mice compared with the control group, which was consistent with mitochondrial structure damage. And HNK treatment made a marked reduction on mtROS and MDA compared with the control group (*p* < 0.05, Figures 4(d) and 4(e)). Inhibition of mitophagy by 3-MA exaggerated the content of mtROS and MDA in the mice which underwent surgical stress up to 7 days (*p* < 0.05, Figures 4(d) and 4(e)).

**Figure 5:**
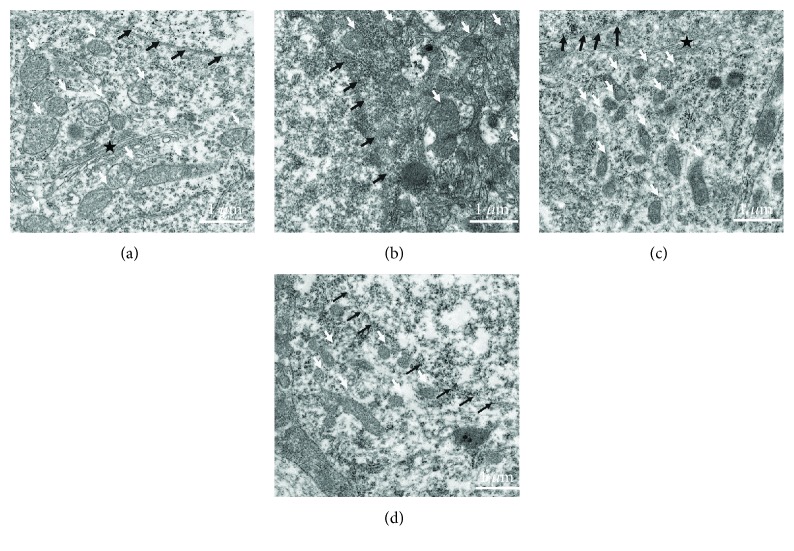
Ultrastructural mitochondrial change in the hippocampus after surgery/sevoflurane exposure: (a) healthy nuclear membrane (black arrowheads), mitochondria (white arrowheads), and cytoplasmic Golgi complexes (black pentagram) in the control group; (b) severe cell damage appeared in the surgery/sevoflurane group: nuclear membrane shrinkage (black arrowheads), mitochondrial matrix appeared darker, structural disorganization of mitochondrial cristae (white arrowheads), and other organelles are vague and difficult to recognize; (c) in the surgery+HNK group, the degree of cellular organelles above were reduced; and (d) in the surgery+HNK+3-MA group, the protective effects of HNK on cellular organelles were reversed (scale bar = 1 *μ*m).

Based on TEM, we observed that surgery under sevoflurane could induce mitochondrial structure damage at postoperative 1 day (Figure 5). Compared to healthy cellular organelles in the control group, severe cell damage appeared in the surgery/sevoflurane group. Nuclear membrane shrinkage, dark mitochondrial matrix, and structural disorganization of the mitochondrial cristae were found in the hippocampus of the surgery/sevoflurane mice. Besides, other organelles are vague and difficult to recognize; HNK ameliorated the destruction of mitochondria in the hippocampus. However, the protective effects were eliminated in the 3-MA intervention group.

These above results suggested that the augmentation of mitophagy by HNK may contribute to the maintenance of mitochondrial quality following surgery/sevoflurane.

### 3.3. HNK Alleviates the Activation of NLRP3 Inflammasome and Microglia in the Hippocampus of Mice Treated by Surgery/Sevoflurane

Accumulation of mitochondrial ROS is one of the triggers of NLRP3 inflammasome activation, which exaggerates the inflammatory response and expedites proinflammatory cytokine release.

**Figure 6:**
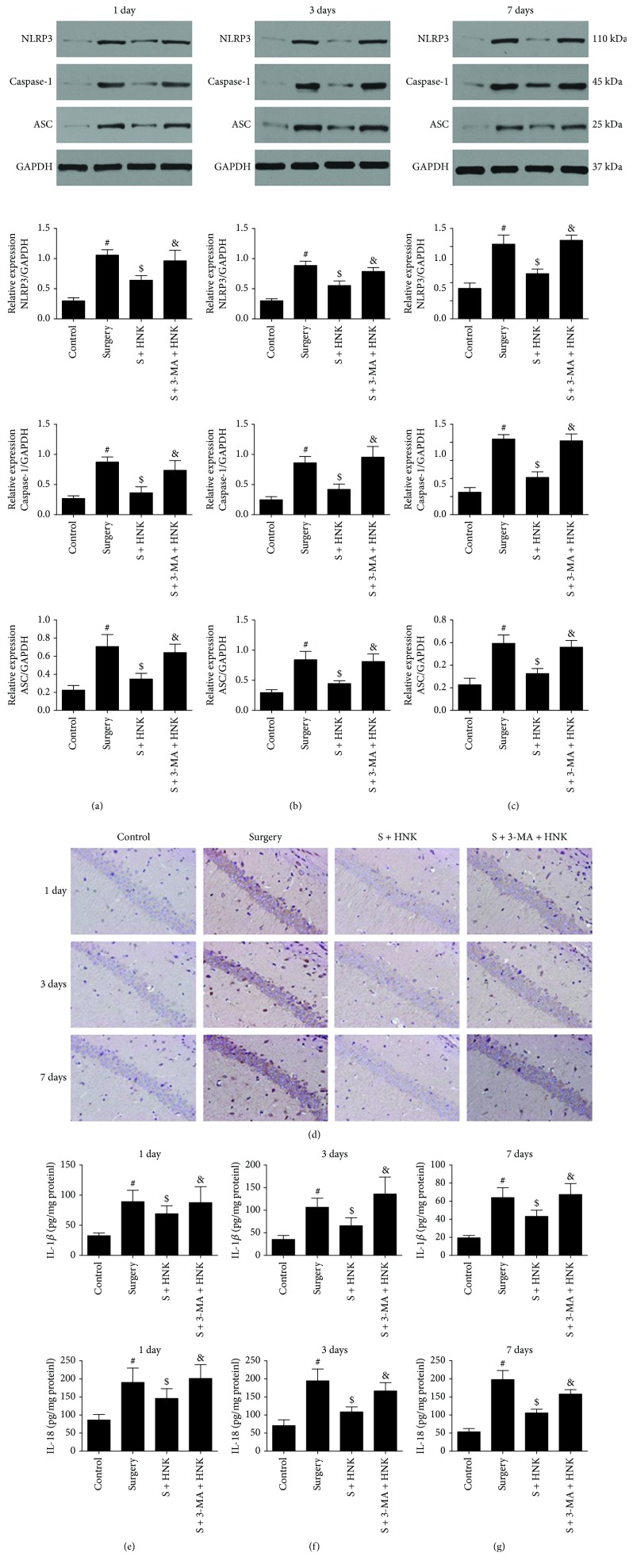
The effects of HNK on NLRP3 inflammasome activation in the hippocampus induced by surgery/sevoflurane. (a–c) The Western blot representative blots of NLRP3, ASC, and Caspase-1 (*n* = 6 per group). (d) The immunohistochemistry of NLRP3 in the hippocampus at 24 h after surgery/sevoflurane. (e–g) The concentration of IL-1*β* and IL-18 in the hippocampus at postoperative 1, 3, and 7 days. The data are presented as the mean ± standard error of the mean for each group. ^#^*p* < 0.05 versus the control group; ^$^*p* < 0.05 versus the surgery+vehicle group; and ^&^*p* < 0.05 versus the surgery+HNK group.

We determined the effects of surgery and HNK and/or 3-MA pretreatment on the expression of NLRP3, ASC, Caspase-1, IL-1, and IL-8 in the hippocampus of the mice on postoperative days 1, 3, and 7 by Western blotting, ELISA, and immunohistochemistry, respectively. Surgery/anesthesia led a marked increase in the expressions of NLRP3, ASC, and Caspase-1 in the hippocampus on postoperative days 1, 3, and 7 compared to the control group (Figures 6(a)–6(d)). These mice that received HNK pretreatment had downregulated the expression of NLRP3, as well as decreased the hippocampal expression of ASC and Caspase-1 on postoperative days 1, 3, and 7. Additionally, 3-MA eradicated the inhibition effects of HNK on NLRP3 inflammasome activation.

The increased secretion of proinflammatory cytokines, including IL-1*β* and IL-18, is in parallel with the NLRP3 inflammasome activation. ELISA analysis showed that the concentration of IL-1*β* and IL-18 increased significantly in the surgery/anesthesia group at postoperative 1, 3, and 7 days, whereas HNK treatment notably suppressed the expressions of IL-1*β* and IL-18 compared with the surgery/anesthesia-induced mice (Figures 6(e)–6(g)). Microglia activation also reflects neuroinflammation. Pretreatment with HNK resulted in a decreased Iba-1 expression on postoperative 1 day, while 3-MA treatment could result in an increased Iba-1 expression compared to the surgery group, which indicated the different activation of microglia (Figure 3(f)).

### 3.4. HNK Suppresses Neuronal Apoptosis in the Hippocampal CA1 and DG Regions of Mice Induced by Surgery/Sevoflurane

**Figure 7:**
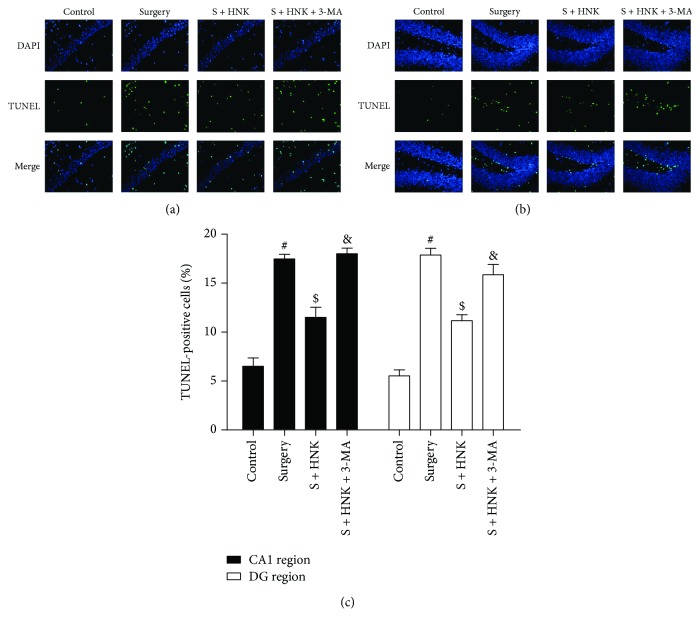
The effects of HNK on neuronal apoptosis in the hippocampal CA1 and DG regions of mice induced by surgery/sevoflurane. (a, b) The representative images showing TUNEL staining in the hippocampal CA1 and DG regions at 24 h after surgery/sevoflurane. The apoptotic cells were detected by TUNEL (green), and the nuclei were detected by DAPI (blue). Scale bar = 50 *μ*m. (c) The percentage of TUNEL-positive cells. The data are presented as the mean ± standard error of the mean for each group (*n* = 3 per group), ^#^*p* < 0.05 versus the control group; ^$^*p* < 0.05 versus the surgery+vehicle group; and ^&^*p* < 0.05 versus the surgery+HNK group.

To reveal whether mitophagy was associated with the neuronal apoptosis, 3-MA was utilized as described above in the surgery/sevoflurane-induced mice. As shown in Figure 7, TUNEL-positive cells were rarely detected in the control group, whereas there were more TUNEL-positive cells in the surgery/sevoflurane group compared to the control group, suggesting that surgery/sevoflurane led to neuronal damage. HNK pretreatment dramatically deceased the neuronal apoptosis in both the hippocampal CA1 and DG regions compared with the surgery/sevoflurane group. However, 3-MA inhibited the neuroprotection of HNK on neuronal apoptosis.

## 4. Discussion

POCD can deteriorate surgery and lead to an induction of morbidity and mortality in patients [26]. Revealing the pathogeny and treatments for POCD are beneficial for the increase of hospitalization comfort and improvement of the long-term outcome. In the current study, we first provided evidence that mitophagy or autophagy was involved in postoperative cognitive impairment induced by surgery/sevoflurane in mice. Honokiol could further induce the mitophagy in surgery/sevoflurane-induced mice. Besides, honokiol inhibited the activation of NLRP3 inflammasome after surgery/sevoflurane treatment and alleviated the neuroinflammation in mice. Pretreatment with honokiol could also preserve neuronal apoptosis and alleviate cognitive decline following surgery/sevoflurane treatment. In addition, 3-MA, an autophagy and mitophagy inhibitor, reversed the neuroprotective effect of HNK on the inhibition of NLRP3 inflammasome activation. These results demonstrated that HNK-mediated mitophagy ameliorates postoperative cognitive impairment induced by surgery/sevoflurane in mice via inhibiting the activation of the NLRP3 inflammasome.

Surgical stress and long duration of inhaled anesthetics can then prompt the activation of immune cells which contributes to neuroinflammation and cognitive change [27, 28]. Without effective intervention, the transformation from acute neuroinflammation to chronic neuroinflammation, which was accompanied by neutrophil infiltration and microglia/macrophage activation, may result in neuronal death, disruption of the blood-brain barrier, and brain edema [26, 29–32]. All the time, anti-neuroinflammatory strategies exhibit potential therapeutic effects against surgery/anesthesia exposure and improve cognitive functions after surgery/anesthesia stress. In recent years, the NLRP3 inflammasome became a research focus in a series of diseases, such as diabetes, neurodegenerative disorders, and brain trauma. Previous studies showed that the MPTP mouse model of Parkinson's disease, traumatic brain injury, cerebral ischemia-reperfusion injury, and isoflurane-inducedhippocampal inflammation can induce the NLRP3 inflammasome activation [6, 8, 33]. Moreover, the inhibition of the NLRP3 inflammasome provided robust neuroprotective response in these pathophysiological processes, which was associated with NLRP3-mediated inflammation and reduction of proinflammatory cytokines. Our findings also suggested that HNK could effectively suppress NLRP3 inflammasome activation and subsequent inflammatory cytokine expression. Immunofluorescence results also showed that trends of microglia activation are correlated with the NLRP3 inflammasome expression in surgery/sevoflurane-induced mice, which is consistent with previous studies. Emerging evidences demonstrated that NLRP3 seems sensitive to the imbalance of cellular homeostasis, so there are a great many of NLRP3 activators, such as low intracellular K^+^ concentration, lysosomal lysis, mitochondrial ROS, or mitochondrial DNA released from damaged mitochondria and Ca^2^ flux [11]. In our studies, we further detected the levels of mitochondrial ROS and MDA, which could reflect the relationships between oxidative stress and the NLRP3 inflammasome. Our results showed that HNK possessed strongly antioxidant capacity which decreased the levels of mtROS and was critical for the reduction of the NLRP3 inflammasome. This study may be the first to exhibit the bridge between oxidative stress and NLRP3-mediated neuroinflammation in postoperative cognitive decline induced by surgery/sevoflurane.

Mitochondria are the primary platforms for energy production and are hypersensitive to the surgical stress. When mitochondrial function is damaged, the overplus of ROS, especially for mitochondrial ROS, is deleterious for the normal physiological state and could cause oxidative stress and inflammation, which are the basis of a wealth of diseases, such as diabetes and cerebral/myocardial ischemia-reperfusion injury [34, 35]. In the view of mitochondrial morphology and biochemical results, we found that HNK effectively blunted the mitochondrial injury (decrease of swelling mitochondria) and the degrees of oxidative stress. These results provided a novel sight for NLRP3 inflammasome activation in postoperative cognitive decline induced by surgery/sevoflurane.

Mitophagy, as a selective autophagy, is a crucial mediator of the degradation of injured mitochondria. Larger numbers of evidence have indicated that autophagy/mitophagy emerges in response to various conditions, such as nutrient depletion, mitochondrial dysfunction, or red blood cell maturation, and has a significant impact on a series of diseases [36]. Lin et al. showed that inhibition of mitophagy could aggravate the neuroinflammation induced by traumatic brain injury. And melatonin enhanced mitophagy through the mTOR signaling pathway, then ameliorated the TBI-triggered neuroinflammation [37]. And not all mitophagy is processed by the Parkin/PINK1 pathway. Studies of the Bnip3/Nix pathway and the Parkin/PINK1 signaling pathway all have a direct connection between defective mitochondria and mitophagy [36]. The difference is that the Parkin/PINK1 pathway depends on the voltage-dependent inhibition in the autophagosome clearance process [38, 39]. And the dysfunction of the Parkin/PINK1 mechanism may lead to defects in mitochondrial morphology, dynamics, and function and result in an imbalance in mitochondrial fusion and fission. However, mitophagy involved in the Bnip3/Nix pathway may be crucial for maintaining the number and the function of mitochondria during cell differentiation and dedifferentiation [40]. In our studies, we found that HNK increased the mitophagy-related proteins, Parkin/PINK1, and protected the abnormal mitochondrial structure (e.g., fragmented cristae and swollen, distorted mitochondrial morphology) in the surgery/sevoflurane model, suggesting that increased mitophagy may eliminate more damaged and dysfunctional mitochondria and may be helpful in reducing the overproduction of ROS. These signaling pathways may be the upstream of NLRP3 inflammasome activation. And different disease models have demonstrated the assumption above. For example, Kim et al. demonstrated that SESN2/sestrin2, an autophagic inducer, prevents sepsis by inducing mitophagy and inhibiting NLRP3 via an increase of unc-51-like kinase 1 protein activity and levels [41]. Consistent with these outcomes, Zhong et al. observed that the NF-*κ*B signaling pathway can translocate the increased cargo receptor p62 to damaged mitochondria, which are recognized by Parkin-dependent ubiquitin and induce mitophagic clearance. The intrinsic regulatory loop “NF-*κ*B-p62-mitophagy” in the macrophage restrains NLRP3 inflammasome activation and maintains homeostasis [42]. Our findings and these studies indicate that mitophagy is one of the self-limiting systems to protect tissues and organs from hyperinflammation and favor tissue repair.

However, it is noted that there are some limitations in our current study. Firstly, in view of behavior tests, we just performed three timepoints (postoperative 1, 3, and 7 days). Due to lack of behavioral data at postoperative 28 days and continuous monitoring within postoperative 24 hours, we failed to understand the fluctuations of cognitive impairment. Further investigation would observe the long-term postoperative cognitive change. Second, in our studies, we just measured the related indicator only in the hippocampus. Several studies also have reported that other brain regions, such as the prefrontal cortex and amygdaloid nucleus, could participate in cognitive function. So, these regions would be investigated in the future. Third, considering the antianxiety property of HNK and our previous study, we cannot exclude the possibility that the change in anxiety levels by HNK hindered the freezing behavior in the fear conditioning test in mice. Finally, due to the expensive price and limited number, we only used 8-week-old female mice but not the aged mice. However, several studies have shown that surgical stress could also induce cognitive change in female mice. And in the future investigation, we will add the observation of the effects of surgery/sevoflurane on postoperative cognitive decline in mice of different sexes and ages.

## 5. Conclusions

Taken together, our study deepened the understanding of the neuroprotective effects of HNK on surgery/sevoflurane-induced postoperative cognitive impairment and detected a novel therapeutic target. Our results indicated that honokiol-mediatedmitophagy ameliorates postoperative cognitive impairment induced by surgery/sevoflurane. This neuroprotective effect may be involved in inhibiting the activation of the NLRP3 inflammasome and suppressing inflammatory responses in the hippocampus.

## Data Availability

The datasets analyzed during the current study are available from the corresponding author on reasonable request.
